# Quantifying the spatial pattern of dialect words spreading from a central population

**DOI:** 10.1098/rsif.2020.0335

**Published:** 2020-07-08

**Authors:** Takuya Takahashi, Yasuo Ihara

**Affiliations:** Department of Biological Sciences, The University of Tokyo, Hongo 7-3-1, Bunkyoku, Tokyo 113-0033, Japan

**Keywords:** cultural evolution, dialect distribution, spatial pattern, geolinguistics, centre–periphery structure

## Abstract

Some dialect words are shared among geographically distant groups of people without close interaction. Such a pattern may indicate the current or past presence of a cultural centre exerting a strong influence on peripheries. For example, concentric distributions of dialect variants in Japan may be explicable by repeated inventions of new variants at Kyoto, the ancient capital, with subsequent outward diffusion. Here we develop a model of linguistic diffusion within a population network to quantify the distribution of variants created at the central population. Equilibrium distributions of word ages are obtained for idealized networks and for a realistic network of Japanese prefectures. Our model successfully replicates the observed pattern, supporting the notion that a centre–periphery social structure underlies the emergence of concentric patterns. Unlike what has previously been claimed, our model indicates that a novelty bias in linguistic transmission is not always necessary to account for the concentric pattern, whereas some bias in the direction of transmission between populations is needed to be consistent with the observed absence of old words near the central population. Our analysis on the realistic network also suggests that the process of linguistic transmission was not much affected by between-prefecture differences in population size.

## 1. Introduction

A dialect is a variant of a language that is spoken by a distinct group of people, where regional dialects may differ from each other in terms of phonology, lexicon, morphology and syntax [1–3]. On the level of linguistic variation within a language family, similarities between languages have been used to reconstruct the phylogenetic relationship among human populations [4–6] based on the premise that populations linguistically more similar to each other are likely to have diverged more recently from an ancestral population (i.e. cultural macroevolution; [7]). Similarly, on the level of dialect variation, much quantitative research has shown that the linguistic distance of each locality is to some extent explained by the geographical distance, although its correlation coefficient varies depending on how geographical distance is measured [8,9]. These studies give impression that the similarity of language reflects the phylogeny of human groups, but it is also often the case that the same dialect variant of a word is documented in phylogenetically distant local groups [10,11], which is likely due to diffusion of words between groups.

On the basis of extensive documentation of Japanese dialects, Yanagita described peculiar geographical distributions of words within the country [10]. In particular, he pointed out that the same dialect variants of the word for snail (*kagyu*) were seen in both ends of the east–west stretch of the land, while they were absent in the middle. Similar patterns of dialect words were found in the nationwide project of Linguistic Atlas of Japan (LAJ) [1], in which words meaning face epitomize this distribution (available at https://mmsrv.ninjal.ac.jp/laj_map/data/laj_map/LAJ_106.pdf). To account for these patterns, Yanagita posited that dialect forms in Japanese may exhibit a concentric distribution centred at Kyoto, the old capital in the middle Japan. According to this theory, new words were repeatedly invented in Kyoto and diffused gradually outward to the periphery, leaving concentric traces. Underlying assumptions are that new words were preferentially adopted by people, perhaps owing to Kyoto's prestige as the capital, and that the diffusion was slow relative to the creation of words, which is plausible given the absence of modern technologies such as television or the Internet.

Concentric distribution of word variants is not unique to Japanese language but occurs in other places where populations are socially or geographically structured into centres and peripheries (hereafter the centre–periphery structure). For example, research based on linguistic atlases of Breton and French languages [3,12] has revealed that several word forms are distributed in a concentric pattern in Lower Brittany, highlighting a profound impact of economically and culturally important towns on the spread of word variants [11]. Despite the ubiquity of similar concentric patterns, most previous studies have merely proposed verbal explanations of the phenomenon without presenting any quantitative analysis. In particular, there is a dearth of mathematical rationale to unveil the underlying factors of the concentric patterns in dialects.

For a mathematical treatment of geographical patterning of dialect variants in the presence of the centre–periphery structure, we need a model considering linguistic influences among multiple groups of people. One commonly used framework is the gravity model [13], in which the mutual influence of two centres (towns, cities, etc.) is assumed to be proportional to the product of their populations and inversely proportional to the squared distance between them. This model predicts that linguistic features first diffuse from city to city, skipping the rural area in between. Kretzschmar [14] used cellular automaton (CA) as a computational model to investigate temporal changes in linguistic features across areas. Fagyal *et al*. [15] developed an agent-based simulation to investigate the language change in a heterogeneous social network, in which highly connected and isolated agents constitute a centre–periphery structure. Burridge [16,17] has recently developed spatially explicit models of linguistic change, borrowing methods from statistical physics. Incorporating demographic data, he demonstrated the spread of words from a city, or densely populated area, to the peripheries. These models provide explanations for interesting linguistic phenomena, including temporal dynamics of dialect boundaries; however, they are silent about the possibility of concentric dialect distribution. This is because these models are designed to deal with a fixed number of pre-existent dialect variants and thus do not allow for repeated inventions of new words in a central population as presupposed by Yanagita [10].

A theoretical study that is more relevant to the current context is by Lizana *et al*. [18], who focused on the proposed concentric distribution of swear words in Japanese dialects, such as *aho* and *baka*, meaning a stupid person [19]. They ran a computer simulation on a two-dimensional lattice that represents the real geography of the Japanese Archipelago, assuming that new words are repeatedly invented in Kyoto and then transmitted to neighbouring regions. A critical assumption was that there is a novelty bias in the transmission of words, so that a newer variant will invade and replace an older variant occupying a lattice site, but not vice versa. The simulation successfully reproduced two empirical features of the swear-word distribution: (i) the same variants are found both to the east and west of Kyoto; and (ii) the geographical band within which a variant is found is broader when it is further from Kyoto. The same research also reported that the absence of the novelty bias results in the disappearance of the concentric pattern.

While Lizana *et al*.'s work [18], which is mostly numerical, has demonstrated that a concentric distribution of words can indeed be formed under a set of reasonable assumptions, a fuller mathematical analysis would shed more light on the processes of linguistic diffusion underlying the observed patterns of linguistic variation. To achieve the latter, this paper develops a mathematical model, assuming a network of populations with a central population from which every word derives, as a simplest representation of the centre–periphery structure. Our model differs from the previous study in three ways. First, we deliberately omit the novelty bias in the transmission of words. Although Lizana *et al*. suggested that the appearance of the concentric pattern was conditional on the presence of the preference for novel words, we show that this assumption is not always necessary for the formation of a concentric distribution. Second, while only one variant occupies each lattice site in Lizana *et al*.'s model, multiple variants can coexist in a single population in our model. The frequency of individuals having a given variant is represented by a real number ranging from zero to one in each population. This assumption seems more realistic because speakers in a single population may use different words, or multiple dialects may be seen in the same group of people. In fact, questionnaire-based research has reported that some respondents answered multiple *aho-baka* expressions prevalent within the same area [19]. Finally, and as a corollary to the second point, we do not define the distance from the central population for each variant. This is because in our formulation each variant may be used in different frequencies in multiple populations, which is unlike Lizana *et al*.'s model. Instead, we track changes in the distribution of word ages in each population. As every word is consecutively invented in the central population, different word age corresponds to a different variant, so we can indirectly deduce the distribution of words by quantifying the spatial pattern of word ages.

In what follows, we will first develop general formulae to calculate the distribution, mean and standard deviation of word age in each population within a network of populations, under the assumption that populations are large (§2). In §3, we apply them to simplistic, schematic networks in order to grasp the general characteristics of the word-age pattern. In particular, we will treat the following idealized networks:

(1) One-dimensional lattice with unidirectional diffusion
(2) One-dimensional lattice with bidirectional diffusion
(3) Two-dimensional lattice
(4) One-dimensional lattice with a barrier
(5) Two-dimensional lattice with a barrier

Section 4 examines the distribution on a more realistic network, based on the network of Japanese prefectures. Also, in electronic supplementary material, we investigate to what extent our model can be applied to smaller populations, in which random cultural drift plays a non-negligible part, using agent-based simulations.

## 2. Theory

### 2.1. Description of the model

We consider transmission of a linguistic trait within and between n+1 populations, $P_{0},\;P_{1},\ldots ,\;P_{n},$ each of which consists of a sufficiently large number of individuals, where the assumption of large population sizes is for the sake of mathematical simplicity. Innovations of words occur only in population $P_{0},$ which we call the central population. In every time step, one novel word is invented and immediately spreads within $P_{0}.$ We treat a polychotomous linguistic trait, such as multiple words meaning the same object, or different pronunciations and intonations for the same word. Thus, an individual can have only one variant at a given time. This is analogous to the one-locus model in population biology. Members of populations other than $P_{0},$ which we call peripheral populations, may obtain a variant by learning socially from an individual in the same or other populations. After social learning, all individuals' variants are updated simultaneously at the beginning of the next time step. We define t=0 as the time when the central population emerges, and transmission starts.

Every single linguistic variant in $P_{k}\;(0\leq k\leq n)$ derives from $P_{0},$ given it was created when t≥0, so we can distinguish the variants by their ages. Let $f_{k}(\rho ,t)$ denote the frequency of the variant aged ρ (≥0) in population $P_{k}$ at time step t, where the age of a variant is measured by the number of time steps elapsed since the variant was created and does not indicate any concrete time unit such as year, decade or generation. We have

2.1 $$\;\;\overset{\infty }{\underset{\rho =0}{\sum }}f_{k}(\rho ,t)=1\;\;\;(0\leq k\leq n).\;$$

Here, 0≤ρ≤t corresponds to the variants that were invented in $P_{0},$ whereas ρ>t represents the ones that had already existed when the central population emerged at t=0.

As for the central population $P_{0},$ the frequencies of word ages are written as

2.2 $$\;f_{0}(\rho ,t)=\left\{\begin{array}{c} 1\;\;\;(\text{if}\;\rho =0)\\ 0\;\;\;(\text{if}\;\rho >0) \end{array}\right.,$$

which means that all individuals in the central population always have the latest variant. In peripheral populations, each individual chooses a role model from whom to learn a linguistic variant. In the choice of role model, a learner first chooses a population to which a potential role model belongs, where the probability with which a learner in $P_{i}$ chooses $P_{j}$ is denoted by $a_{ij}$ (≥0) (1≤i≤n,0≤j≤n), and then chooses a role model from all individuals in the chosen population with equal probability. Since the population is sufficiently large for stochastic effects to be negligible, we can deterministically obtain the following recursive formula as regard to frequencies in the peripheral populations:

2.3 $$f_{k}(\rho ,t)=\overset{n}{\underset{\;j=0}{\sum }}a_{kj}f_{j}(\rho -1,\;t-1)\;\;\;(1\leq k\leq n).$$

Note that $f_{k}(\rho ,t)$ and $f_{j}(\rho -1,\;t-1)$ represent the frequencies of the same variant in different populations at different time steps. We will refer to $a_{ij}$ as the transmission rate from $P_{j}$ to $P_{i},$ which may depend on the geographical proximity, population sizes or social prestige of the populations. In particular, $a_{ii}$ represents the transmission rate within one population, indicating to what extent the word stays the same between time steps. The transmission rate to $P_{0}$ is not defined because the central population does not learn from other populations by assumption. Note that the transmission rates are the same for all variants regardless of their ages ρ or frequencies. In other words, transmission of words is assumed to be unbiased, and novelty bias or frequency bias (e.g. conformity to the local majority) is absent in this model.

The transmission rates characterize the topological structure of the network. Our model considers arbitrary networks in which words created in $P_{0}$ can reach all $P_{k}(1\leq k\leq n).$

The definition of transmission rate gives

2.4 $$\;\overset{n}{\underset{\;j=0}{\sum }}a_{kj}=1.\;$$

### 2.2. Distribution of word age

Defining $f(\rho ,t)={\left(\;f_{1}(\rho ,t)\ldots f_{n}(\rho ,t)\right)}^{T}$ and $A=\left(\begin{array}{ccc} a_{11} & \cdots & a_{1n}\\ \vdots & \ddots & \vdots \\ a_{n1} & \cdots & a_{nn} \end{array}\right),$ we have the distribution of age frequency in the *n* peripheral populations:

2.5 $$f(\rho ,t)=\left\{\begin{array}{c} 0\;\;\;\;\;\;\;\;\;\;\;\;\;\;\;\;\;\;(\rho =0)\\ A^{\rho -1}\left(\begin{array}{c} a_{10}\\ \vdots \\ a_{n0} \end{array}\right)\;\;\;\;\;\;\;(1\leq \rho \leq t)\\ A^{t}f(\rho -t,0)\;\;\;(t<\rho ) \end{array}\right.$$

where f(ρ,0) is the initial distribution of word ages, defined for any ρ>0, in *n* peripheral populations. Let $r(t)={\left(r_{1}(t)\cdots r_{n}(t)\right)}^{T}$ represent the vector whose kth element corresponds to the mean word ages in $P_{k}.$ We have

2.6 $$\begin{array}{rl} \;r(t) & =\overset{\infty }{\underset{\rho =0}{\sum }}\rho f(\rho ,t)\\ & =A^{t}\left(r(0)-{\left(E-A\right)}^{-1}\left(\begin{array}{c} 1\\ \vdots \\ 1 \end{array}\right)\right)+{\left(E-A\right)}^{-1}\left(\begin{array}{c} 1\\ \vdots \\ 1 \end{array}\right). \end{array}$$

For the equilibrium state, we have

2.7 $$\;r(\infty )={\left(E-A\right)}^{-1}\left(\begin{array}{c} 1\\ \vdots \\ 1 \end{array}\right),$$

where E represents *n*-dimensional identity matrix. For the derivation of (2.5), (2.6) and (2.7), see electronic supplementary material.

A measure of linguistic diversity within the population is the standard deviation (s.d.) of word age. Let $\sigma_{k}(t)$ denote the standard deviation of word age in population $P_{k}$ at time step t. We can also calculate the equilibrium standard deviation of word age within population, $\sigma (\infty )={\left(\sigma_{1}(\infty )\cdots \sigma_{n}(\infty )\right)}^{T}$ (electronic supplementary material). In addition, we introduce another diversity measure $H_{k}(t)$ as follows:

2.8 $$H_{k}(t)=1-\overset{\infty }{\underset{\rho =0}{\sum }}f_{k}{\left(\rho ,t\right)}^{2}.$$

Here, $H_{k}(t)$ is the heterozygosity of the words in $P_{k},$ or the probability that two randomly sampled variants are not identical, which is analogous to the genetic heterozygosity at a single locus. Whereas $\sigma_{k}(t)$ is used to deduce how words are quantitatively diverse in a population, $H_{k}(t)$ only considers the identity of variants. In computing the infinite series in (2.8), we take summation over ρ from zero to a sufficiently large integer called the ‘cut-off value’. We choose this value so that $f_{k}(\rho ,t)$ is negligibly small for every ρ that is larger than the cut-off.

### 2.3. Analytically tractable cases

**Figure 1.**
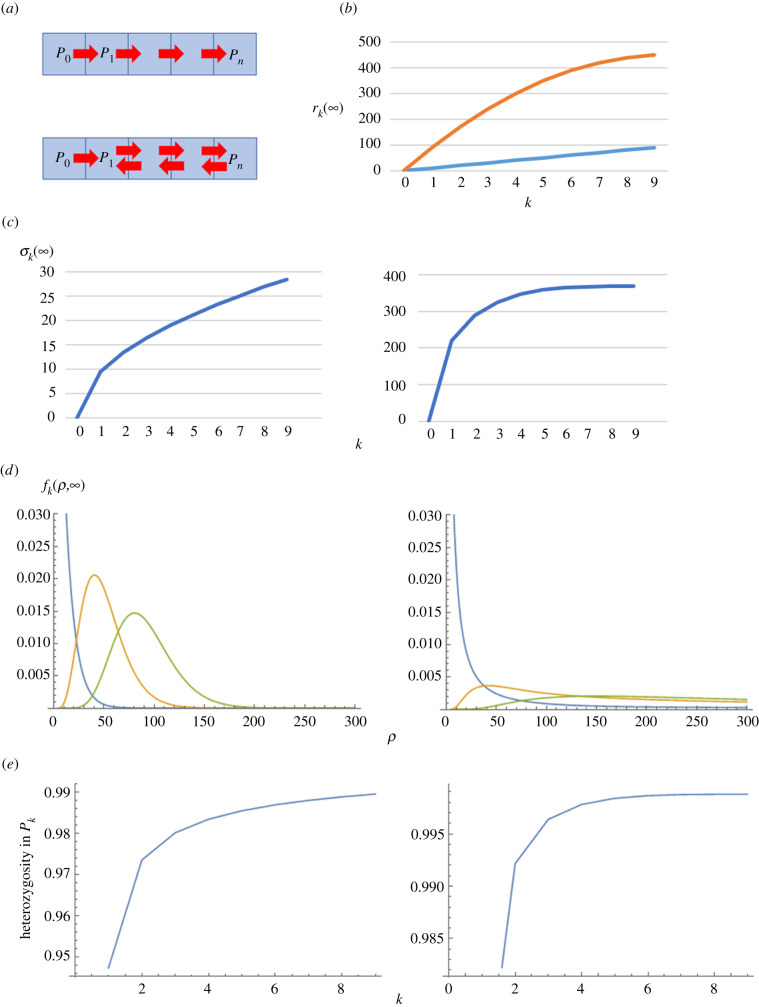
(*a*) A schematic of cultural diffusion in a one-dimensional chain of populations (*n* = 4). The arrows indicate the presence and direction of word transmission from one population to an adjacent population in the case of (above) unidirectional and (below) bidirectional transmission. Note that recursive arrows (loops) are omitted in this panel. (*b*) The mean word age at equilibrium in a chain of populations. The horizontal and vertical axes represent the distance from the central population and the mean word age in each population, respectively. The blue and orange lines represent the unidirectional and bidirectional transmission models, respectively. Parameter values: *a* = 0.1, *n* = 9 (total number of populations: 10). (*c*) The standard deviation of word age at equilibrium in a chain of populations. The horizontal and vertical axes represent the distance from the central population and the standard deviation of word age in each population, respectively, for (left) unidirectional and (right) bidirectional transmission. Parameter values: *a* = 0.1, n=9 (total number of populations: 10). (*d*) Frequency distribution of word age ρ at equilibrium in each population. The blue, orange and green lines represent the populations $P_{1}$, $P_{5}$ and $P_{9}$, respectively, for (left) unidirectional and (right) bidirectional transmission. Parameter values: a=0.1, n=9 (total number of populations: 10). (*e*) Heterozygosity of words in each population with unidirectional (left) and bidirectional transmission (right). Parameter values: a=0.1, n=9  (total number of populations: 10), and cut-off value ρ=1000 and  10000 for unidirectional and bidirectional transmission, respectively.

To provide a further mathematical analysis, we focus on the case when the transmission rate from one population to another is either *a* or 0; that is, $a_{ij}=a>0$ (const.) for some combinations of transmitting and receiving populations and $a_{ij}=0$ for others. Note that this is assumed throughout the rest of this paper unless otherwise stipulated. Suppose further that populations $P_{0},P_{1},\ldots ,\;\text{and}\;P_{n}$ are aligned in this order to form a one-dimensional chain, so that the central population is situated on an edge (figure 1*a*). We consider the following two cases.

First, when transmission is unidirectional from $P_{j}$ to $P_{\;j+1}(0\leq j\leq n-1)$ so that words diffuse toward populations farther from the central population, the transmission matrix is given by

2.9 $$A=\left(\begin{array}{cccc} 1-a & & & \\ a & 1-a & & \\ & \ddots & \ddots & \\ & & a & 1-a \end{array}\right),$$

where zero elements are omitted for the sake of notational simplicity. Based on the matrix, we obtain

2.10a $$r_{k}(\infty )=\frac{k}{a},$$

and

2.10b $$\sigma_{k}(\infty )=\sqrt{\frac{k}{a}\left(\frac{1}{a}-1\right)}.$$

Detailed derivation of (2.10*a*) and (2.10*b*) is given in the electronic supplementary material. These expressions show that words are on average older and more diverse in populations that are located further from the central population (figure 1*b*,*c*).

Secondly, we consider the case of bidirectional diffusion. Words are transmitted bidirectionally between adjacent populations with the exception of $P_{0},$ to which transmission from other populations does not occur. Transmission matrix is written as

2.11 $$A=\left(\begin{array}{cccc} 1-2a & a & & \\ a & \ddots & \ddots & \\ & \ddots & 1-2a & a\\ & & a & 1-a \end{array}\right).$$

The mean and standard deviation of word ages at equilibrium are calculated as follows:

2.12a $$r_{k}(\infty )=\frac{k}{2a}(2n-k+1),$$

and

2.12b $$\begin{array}{rl} & \sigma_{k}(\infty )\\ & =\sqrt{\frac{k}{6a^{2}}(2n-k+1)(2n^{2}-2nk+k^{2}+2n-k+1)-\frac{k}{2a}(2n-k+1)}. \end{array}$$

Again, see electronic supplementary material for more detailed derivation. As in the unidirectional model, $r_{k}(\infty )$ and$\;\sigma_{k}(\infty )$ increase with *k* (figure 1*b*). In addition, they also increase with *n*, which means that word age depends not only on the distance from the central population, but on the length of the population chain. In the bidirectional model, old variants can diffuse from remote populations to ones that are nearer to the central population, so it is natural that words become on average older when the chain of populations is longer.

Figure 1*d* depicts the equilibrium distribution of word age within the same population. In both unidirectional and bidirectional transmission, there is a peak of word age in every population. While old variants are extremely rare in the case of unidirectional transmission, they are maintained at a relatively high frequency with bidirectional transmission. This is because with bidirectional transmission old variants come in not only from more central, but also from more peripheral neighbours, and hence are maintained in peripheries for a long time. Also, heterozygosity of variants increases with *k* for both unidirectional and bidirectional transmission (figure 1*e*) and is larger in bidirectional transmission. It is therefore suggested that the amount of polymorphism is larger under the condition of bidirectional transmission, which is because old words are maintained in the populations.

## 3. Numerical analysis on schematic networks

For less simplified cases, we can numerically obtain the mean and standard deviation of word age at equilibrium. In this section, we describe two such examples.

### 3.1. Two-dimensional diffusion

We now allow bidirectional diffusion for both horizontal and vertical directions in the m×l rectangle of populations. Unlike in the previous one-dimensional model, the central population is not necessarily situated at a corner or edge of the rectangle.

**Figure 2.**
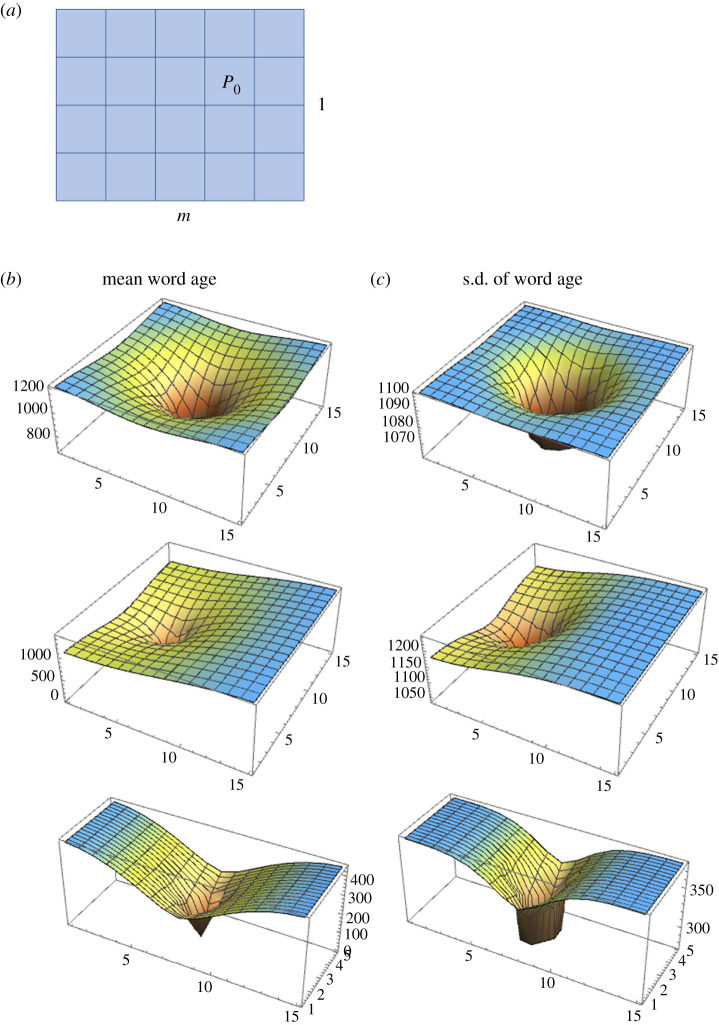
(*a*) A schematic of the two-dimensional diffusion model. Bidirectional cultural diffusion occurs between horizontally or vertically neighbouring populations. In this example, population at (4, 3) plays the role of the central population (denoted by $P_{0}$). Parameter values: m=5, l=4. (*b*) Mean word age at equilibrium in the two-dimensional diffusion model. (Top) m=l=15 and $P_{0}$ is located at (8, 8). (Middle) m=l=15 and $P_{0}$ is assigned to a population not at the centre of the square, specifically, (5, 8). (Bottom) m=15 and l=5 and $P_{0}$ is at (8, 3). (*c*) Standard deviation of word age at equilibrium in the two-dimensional diffusion model. The shape of the rectangle and the location of the central population are the same as in (*b*).

Figure 2*b*,*c* shows 3D plots of the mean, $r_{k}(\infty ),$ and standard deviation, $v_{k}(\infty ),$ of word age at equilibrium over the m×l rectangle of populations. As anticipated, the mean and standard deviation are smallest at the central population and increase with increasing distance from the centre in all cases. Beyond this overall similarity, however, the precise pattern of increase depends on the position of the central population and the shape of the rectangle. First, consider the case when m=l holds, so that populations form a square, and $P_{0}$ is at the centre of the square, ((m+1)/2,(m+1)/2), assuming *m* and *l* as an odd number. In this case, the changes in the mean and standard deviation of word age are symmetric in all four directions (see the top row of figure 2*b*,*c*). Second, in contrast, when $P_{0}$ is placed closer to one of the four sides of the square, the mean and standard deviation of word age increases less rapidly toward that side than toward the opposite side (see the middle row in figure 2*b*,*c*), as a result of which the mean and standard deviation of word age exhibit asymmetric contour lines. Third, when either the horizontal or vertical side of the rectangle is longer than the other (m≠l), the changes in the mean and standard deviation of word age are faster along the longer side than along the shorter side (see the bottom row in figure 2*b*,*c*).

### 3.2. Effect of a natural or cultural barrier

We have so far assumed a constant rate of transmission among populations, but the degree of their interdependence is changeable depending on geographical factors. Here we consider the presence of a barrier which inhibits human interactions and linguistic transmission for some geographical or cultural reason (e.g. mountains, deserted area, culturally conservative population, prohibition of movement, etc.).

First, as the baseline model, we adopt the one-dimensional bidirectional diffusion of words as discussed earlier. Two consecutive populations $P_{h}$ and $P_{h+1}$ are separated by a barrier (e.g. river, mountain, etc.), and we denote the transmission rate between the two populations by *b*. Assuming 0 < *b* < *a*, transmission is weaker between these populations than in other pairs of neighbouring populations.

**Figure 3.**
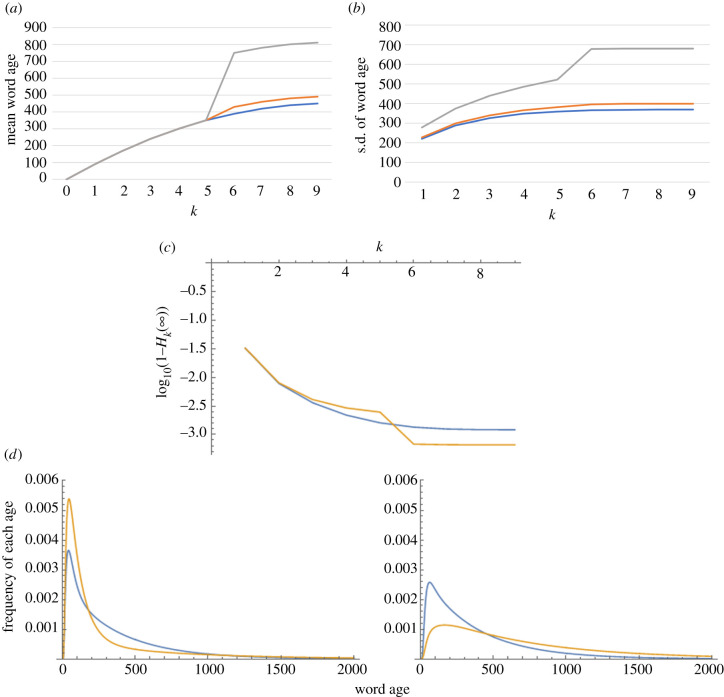
Bidirectional transmission in a chain of population with a barrier. The barrier divides populations $P_{5}$ and $P_{6}$ (h=5). Parameter values: n=9, a=0.1. (*a*) The mean word age at equilibrium when (blue) b=0.1 (i.e. no barrier), (orange) b=0.05, and (grey) b=0.01. The three curves are overlapped when k≤5. (*b*) The standard deviation in word age at equilibrium when (blue) b=0.1 (i.e. no barrier), (orange) b=0.05, and (grey) b=0.01. (*c*) The heterozygosity of variants in each population with cut-off value ρ=10 000 when (blue) b=0.1 (i.e. no barrier) and (orange) b=0.01. For the sake of presentation, the vertical axis shows $\log_{10}(1-H_{k}(\infty )),$ which decreases as the heterozygosity increases. (*d*) Equilibrium frequency distribution of word ages in (left) $P_{5}$ and (right) $P_{6}$ when (blue) b=0.1 (i.e. no barrier) and (orange) b=0.01.

As suggested by figure 3*a*, $r_{k}(\infty )$ becomes larger in the presence of a barrier in populations for which h<k holds, which means that the mean word age at equilibrium becomes older in populations beyond the barrier (from the perspective of the central population). Interestingly, $r_{k}(\infty )$ is not affected in the near side of the barrier (i.e. k≤h) (figure 3*a*), even though variants diffuse in both directions and thus the barrier is expected to have an impact on all populations. As for the diversity estimators, the standard deviation of word age becomes larger on both sides of the barrier (figure 3*b*). Conversely, heterozygosity $H_{k}(\infty )$ increases where h<k and decreases where k≤h (figure 3*c*).

The results are interpreted as follows. Since a barrier inhibits the transmission of novel variants created in $P_{0}$ to remote populations, it is straightforward that $r_{k}(\infty )$ becomes larger in populations farther than $P_{h}$. Where k≤h, the matter is more complicated. In fact, the existence of barrier affects the word age of the near populations in two ways. On the one hand, a barrier makes words in remote populations even older, which results in the influx of old variants into the near populations. On the other hand, as transmission from remote populations is partially insulated, the near populations receive a relatively smaller number of old variants. It seems that these opposite effects are cancelled out, and the mean word age stays unchanged in $P_{k}(k\leq h).$ This interpretation is consistent with the finding that in the presence of a barrier, extremely old variants are maintained at low frequencies in $P_{k}(k\leq h)$ (figure 3*d*), so $\sigma_{k}(\infty )$ increases between the central population and the barrier (figure 3*b*). However, as the number of new words increases significantly, the heterozygosity drops in the near populations (figure 3*c*). In conclusion, the presence of a barrier exerts the opposite influences on the two diversity estimators in populations between the central population and the barrier.

**Figure 4.**
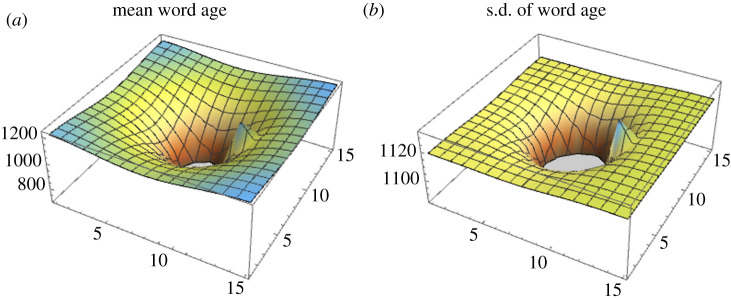
Effect of a barrier in the two-dimensional diffusion model. Populations form a 15×15 square, with the central population being at (8, 8) and a barrier at (11, 8). (*a*) The mean word age and (*b*) the standard deviation in word age. Parameter value: b=0.001.

Secondly, we consider the barrier based on the two-dimensional diffusion model. One of the two-dimensionally arranged populations is an isolated barrier $(P_{h}),$ the transmission to/from which occurs at the rate b(<a). Figure 4 indicates that $r_{k}(\infty )$ becomes smaller between $P_{0}$ and $P_{h},$ and larger on the other side of the barrier. Unlike the one-dimensional model, $P_{h}$ marks a peak of $r_{k}(\infty )$ and $\sigma_{k}(\infty )$ for small values of b. As populations are aligned in a two-dimensional shape, words can be transmitted via multiple pathways, and as a consequence diffusion can detour the barrier. For this reason, the existence of a barrier has less impact on remote populations than in the one-dimensional case.

## 4. Numerical analysis on realistic networks

We have so far analysed populations arranged in a chain or lattice. In this section, we consider an extended model that incorporates a more realistic network of populations reflecting the geography and demography of Japanese prefectures to examine the case of concentric dialect distributions centred at Kyoto.

### 4.1. Adaptation of the model to the network of prefectures

To reflect the geographical features of the Japanese Archipelago, we regard 46 Japanese prefectures except Okinawa as $P_{0},\ldots ,P_{45}$ of our model. We exclude Okinawa because this prefecture is geographically and was historically isolated from other parts of Japan. On the network of 46 prefectures, we regard Kyoto prefecture as the centre, $P_{0},$ from which every linguistic variant derives.

One typical method for modelling the linguistic diffusion on a network of cities is to use the *gravity model*, in which the extent of interaction between two cities is assumed to be proportional to the product of their population sizes and the inversed square of the distance in between [8,10]. However, since this assumption would always give $a_{ii}=1$ in our model, we instead follow Burridge [11] to incorporate a modified gravity model, or the *interaction density*, $\varphi_{ij},$ which is defined as the time people in $P_{i}$ spend interacting with speakers in $P_{j}.$ Here, we adapt his eqn (2.3) to our model:

4.1 $$\varphi_{ij}=\frac{\pi_{i}\pi_{j}}{1+d_{ij}^{2}/\gamma^{2}},$$

where $d_{ij}$ is the distance between $P_{i}$ and $P_{j},$ and $\pi_{i}$ denotes the population size of $P_{i}.$ Constant γ represents the half-decay distance, that is, the distance at which the interaction density is halved relative to that within the same node, where words tend to spread farther when γ is larger. As with Newton's Law of gravity, (4.1) has a long algebraic tail. Geolinguistics has been adopting a variety of measures for geographical distance, such as Euclidean distance [9], great-circle distance (shortest distance on a sphere surface) [8], travel distance [8,9] and railway distance [20]. Here, we use the great-circle distance between prefectural government offices (buildings), summarized in [21]. We use the population data of each prefecture surveyed in 2018 [22]. Although the population size was different during the time of dialect diffusion, as we shall discuss later (see (4.3)), our model depends on the ratio of population sizes, so the modern population size seems to be a good proxy assuming that all the populations grew at a uniform rate.

In this framework, however, we simultaneously observe the effects of both the population sizes of the prefectures and the topological structure of the network. To discuss these two effects separately, we additionally examine a population-independent model, in which case the interaction density, given by

4.2 $$\;\varphi_{ij}=\frac{1}{1+d_{ij}^{2}/\gamma^{2}},$$

is uniquely dependent on the distances of prefectures, irrespective of their population sizes.

Since $a_{ij}$ represents the probability that a person in $P_{i}$ learns a word from a role model in $P_{j},$ it is natural that $a_{ij}$ be given as the interaction density between $P_{i}$ and $P_{j}$ divided by the total amount of interaction (s)he experiences. Therefore, we have

4.3 $$a_{ij}=\frac{\varphi_{ij}}{\sum_{l=0}^{45}\varphi_{il}}.\;$$

In the case of population-dependent interaction given by (4.1), transmission rate $a_{ij}$ is proportional to the population size of $P_{j}$ (the transmitting prefecture), while it decreases as the population size of $P_{i}$ (the receiving prefecture) increases. Based on the model, we calculate numerically the mean word age in each prefecture at equilibrium.

### 4.2. Word age at equilibrium

**Figure 5.**
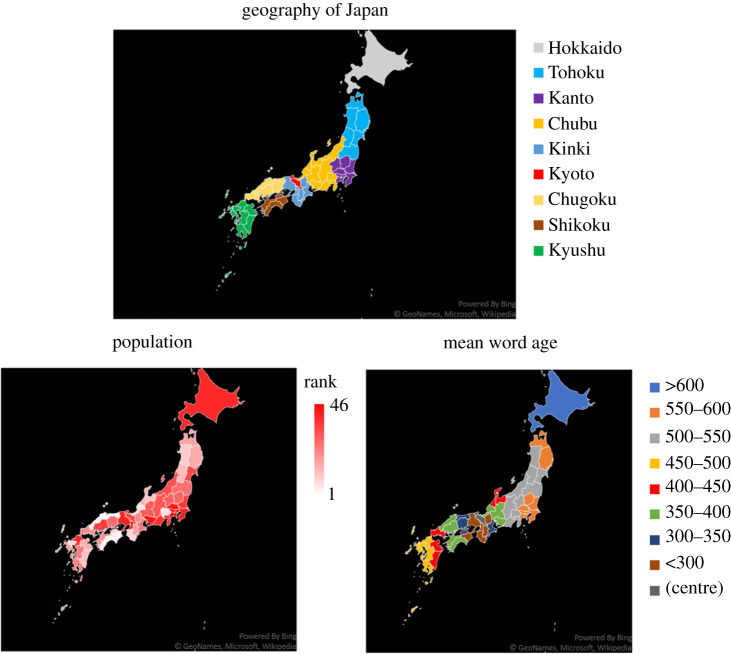
Population-dependent diffusion on a network of Japanese prefectures. (Above) Regions of Japan. Kyoto is coloured red, and Okinawa is not shown on this map. (Bottom-left) Rank order of the population size in each prefecture, the least and most populated prefectures being 1 and 46, respectively. (Bottom-right) Mean word age at equilibrium in each population on the basis of equation (4.1). Parameter value: γ=10 km.

Using equation (4.1), we examine the case in which the interaction density and the transmission rate are proportional to the product of the population sizes of the prefectures. Figure 5 suggests that words become on average older with the distance from Kyoto, but prefectures to the west of Kyoto tend to contain newer words compared to the eastern prefectures located at the same distance from Kyoto. The distribution, therefore, is not symmetric, and words diffuse westward more rapidly than eastward. In this example, in which γ is set to 10 km, there are two separated regions in the east side having similar word ages of between 550 and 600, namely, around Tokyo and the northern Tohoku area. Qualitatively similar results were obtained for γ=20  and  50  km (see electronic supplementary material).

**Figure 6.**
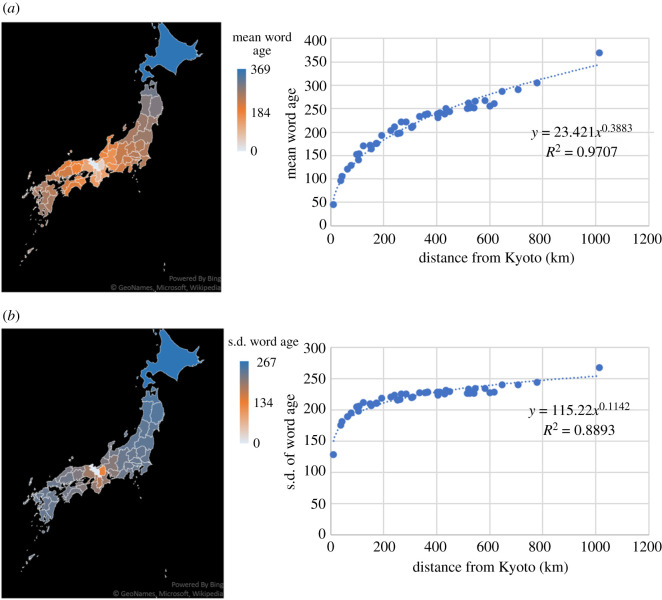
Population-independent diffusion on a network of Japanese prefectures, calculated by (4.2). (*a*) (Left) Equilibrium mean word age in each prefecture. (Right) Equilibrium mean word age as a function of distance from Kyoto. (*b*) (Left) Equilibrium standard deviation of word age in each prefecture. (Right) Equilibrium standard deviation of word age as a function of distance from Kyoto. Parameter value: γ=10 km.

On the other hand, considering the population-independent transmission rate represented by (4.2), words become older almost symmetrically in both sides of Kyoto (figure 6). We also find a decelerating rate of change in mean word age with distance from the centre, indicating that the same word occupies more extensive areas as it goes farther away from the centre. These two features are in concordance with the case of one-dimensional bidirectional diffusion (figure 1*b*).

Comparison of figures 5 and 6 leads to the conclusion that the asymmetric distribution of mean word age in figure 5 is attributable to the heterogeneity in population size. Since transmission rate $a_{ij}$ is proportional to the population of $P_{j}$ (i.e. the prefecture to which the role model belongs) in (4.1), the population-dependent model assumes that people in a highly populated community are likely to learn a word within their own community, delaying the entry of newer words, and as a consequence play a role as a conservative ‘barrier’. Since the Tokyo area harbours an especially large population, the relatively slow diffusion of words into East Japan as predicted in figure 5 may well be interpreted as resulting from hindered diffusion of novel variants from Kyoto into this region.

## 5. Discussion

To understand the emergence of geographical patterns in linguistic variants and the underlying process of diffusion in the presence of a centre–periphery social structure, we have developed a model of linguistic diffusion between populations distributed over space. Using the model, we have quantified the expected frequency distribution of variants, mean and standard deviation of word ages, and amount of linguistic variation in each population. Implications from our main analysis are summarized as follows. First, the mean word age of a given population is expected to increase with its distance from the central population. This indicates that the emergence of a concentric word distribution such as documented in Japan [10,18] and France [3,12] can at least partially be explicable by the presence of a centre–periphery structure. Secondly, difference in the mean word ages between two adjacent populations tends to be highest near the central population and decreases with the distance from it. This finding is in accord with the observed geographical distribution of swear words in Japanese [18], which further supports the hypothesis that the centre–periphery structure underlies the concentric distribution of word variants. Thirdly, even in a population with a relatively low mean word age, old variants are expected to be maintained at a considerable frequency (figure 1*d* right). While this result implies the strong persistence of old words, we have been unable to find empirical support for this prediction. The only exception to the second and third rules is found in one-dimensional unidirectional diffusion, which approximates the case when populations are hierarchically organized in a way that populations closer to the centre bear higher social status. In this case, word age increases lineally in proportion to the distance from the central population (figure 1*b*) and old variants are almost eliminated at equilibrium (figure 1*d*). Therefore, the fact that our third prediction does not receive empirical support may mean that the linguistic diffusion at the time when the concentric distribution was created was only partially bidirectional being biased in favour of the direction from the centre to the periphery.

Lizana *et al*.'s remark [18] on the distribution of swear words is twofold: swear words are arranged in a concentric shape (i.e. concentric distribution of words), and the spatial interval between adjacent words increases with the distance from Kyoto (i.e. extended interval of words). In our analysis, the former is seen most clearly in two-dimensional diffusion with the central population situated at the centre of a lattice. The average word age increases with the distance from the central population, reflecting the fact that newly invented words are prevalent near Kyoto and older words are gradually pushed outward. Strictly speaking, however, the result contradicts the alleged observation in the way that our model produces a mixture of several variants in the periphery, instead of an array of gradually older variants which distinctively dominate each area. The latter feature of the observed distribution is manifested as the decreasing rate of difference in the mean word ages between neighbouring populations. Intuitively, our model predicts that moving outward from the central population, one will initially encounter a drastic linguistic change within a short distance, but the change will be decelerated as moving farther away from the centre. It should be noted, however, that since our model permits the coexistence of multiple variants in the same population, discrete ‘boundaries’ or ‘intervals’ of words cannot be defined. In this regard, therefore, it is difficult to compare Lizana *et al*.'s and our results quantitatively.

The outcome of our extended model that assumes population-dependent cultural diffusion is qualitatively different from the outcome of the main model in that the former predicts a geographically asymmetric word distribution. In particular, our analysis on a network of populations incorporating geographical and demographic characteristics of the present-day Japan does not replicate the observed pattern, where the same dialect variants are used in the east and west ends of the country [6,15]. Since those models that successfully replicate the observed pattern (i.e. ours and Lizana *et al*.'s [18]) assume population-independent cultural diffusion, it is suggested that the historical word diffusion in Japan may have occurred in a population-independent manner.

While we do not explicitly incorporate the novelty-biased transmission as assumed in Lizana *et al*.'s simulation work [18], the same kind of bias is in effect considered in our analysis of one-dimensional unidirectional transmission. Unidirectional transmission may occur when individuals prefer words coming in from the direction of central population, which is tantamount to a bias toward newly invented variants. In contrast to Lizana *et al*., however, our result of unidirectional transmission does not support the extended interval of words. Our analysis suggests that the extended interval of words is expected only with bidirectional transmission, which corresponds to the absence of a novelty-bias. In addition, Lizana *et al*. argues that concentric distribution of words appears only when people prefer new variants, but our model shows this pattern without a novelty bias. Therefore, there is a clear discrepancy between our and Lizana *et al*.'s result in terms of novelty bias. Note that although Lizana *et al*. [18] claims the feature of extended interval of swear word variants in remote areas, we could not find other clear examples of this characteristic [1]. It may be that the increase in spatial interval between adjacent words is usually so subtle that can hardly be detected empirically.

Our prediction from the two-dimensional diffusion model that old words persist in populations around the centre is at odds with the empirical observation that dialect words documented in the east and west peripheries of Japan, which were supposedly created in the centre in the past, are no longer found in Kyoto [1]. The observation is more similar to the outcome of our one-dimensional unidirectional diffusion model. This may indicate that the linguistic diffusion from Kyoto was not strictly bidirectional as our bidirectional model assumes and was at least partially unidirectional. As mentioned above, people's preference for novelty or prestige may have an effect similar to unidirectional diffusion.

The analysis of rectangular-shaped population implies that words tend to be older in the longer side of the land. Since the Japanese Archipelago is long and narrow from southwest to northeast, the shape of word distribution may be elliptic rather than circular. From Kyoto, the distance to the seashore is much shorter in the south and north than east and west directions. Thus, our model predicts that word variants may be older in the west and east of Kyoto and relatively new in northern and southern part. Testing this prediction would be interesting if relevant data are available.

Our two measures of linguistic diversity, $\sigma_{k}$ and $H_{k},$ partially contradict each other, in particular in the analysis of the barrier. When transmission is insulated in one-dimensional bidirectional diffusion, the standard deviation of word age, $\sigma_{k}$ increases in populations located between the central population and the barrier, while the heterozygosity, $H_{k},$ decreases in the same populations. Therefore, the effect of barrier on these populations seems equivocal (i.e. linguistic diversity is indicated to either increase or decrease, depending on how it is measured). Which of the two measures is more appropriate depends on the nature of the linguistic trait of interest. If the relevant trait is a quantitative trait that is subject to only gradual and unidirectional changes of the trait value (e.g. different accents of the same word), the standard deviation of age would represent the polymorphism, because the time of creation directly corresponds to the amount of difference. On the other hand, if the trait of interest is a qualitative trait subject to discrete changes (e.g. synonyms with different etymology), the time of creation does not provide information of the variants, and thus the heterozygosity is the better measure of the polymorphism.

We discuss possible applications of the present study. Although we have focused on the transmission of dialects as a test case, our model may be applicable to other socially transmitted behaviours or *culture* originating from a culturally influential population. The transmission of human cultures is extensively studied in the discipline of cultural evolution [7,23,24], and spatial patterns of cultural traits have been treated using phylogenetic approaches [25–30]. As well as the transmission of dialects, some populations play a greater role in the transmission of culture in general. For example, observed geographical patterns in the prevalence of the ‘hinoeuma’ superstition within the Japanese Archipelago is better explained by considering the presence of a cultural centre, or a single prefecture of prominent cultural influence [31]. Another theoretical study investigated the spread of information in conjunction with the appearance of cultural centre [32]. Our model can be extended beyond the linguistic traits and can treat the spatial pattern of other culturally transmit traits which spread from a single population. For example, archaeological records suggest that stone weapons and burial goods were transmitted from the Eurasian Continent to the Japanese mainland via the Korean peninsula and Kita-Kyushu areas and eventually diffused to the eastern part of Japan [33]. In this case, these areas can be seen as the cultural centre, from which these archaeological traits derive. It is intriguing to investigate whether our model is consistent with empirical archaeological data.

We discuss the limitations of our model and present suggestions for future work. Firstly, we have assumed for mathematical convenience that new words or dialect variants are invented exclusively in the central population and transmitted to other populations without any modification. While the reality is less simple than that, changes in the model outcome caused by relaxing the assumption would be rather predictable. For example, if the central population is not always filled with the latest variant, or if the periphery can also influence the centre, word age will presumably increase because a relatively smaller number of novel variants will diffuse toward periphery.

Secondly, our assumption is that only the central population creates new word variants. This is one of the simplest representations of the centre–periphery structure that we consider as a common feature underlying the observed cases of concentric word distribution. While we show that a concentric distribution is indeed predicted under this assumption, whether this holds true when the peripheral populations sometimes invent or modify words is yet to be investigated. It would be more realistic to integrate multiple centres which create new words at different rates depending on their respective prestige and population size. Such an investigation, however, would require a completely new mathematical framework, which is able to keep track of multiple variants created in the same time step in different populations, and thus is beyond the scope of the present study.

Thirdly, while our analysis on a realistic network reflecting the distance and population size of Japanese prefectures enables a close comparison of expected and observed word distributions, it is still difficult to perform any rigorous quantitative test. Such a test would require estimates of the ages of word forms, which are not available in any linguistic atlases. Nonetheless, our model proves to be useful in inferring the mode of cultural diffusion during the formation of a concentric distribution.

Finally, even though we have considered the word ages in each population, age does not necessarily correspond to the degree of qualitative difference between linguistic variants. To analyse the difference explicitly, we need to model how rapidly words change over generations in the central population. If the latest variant is almost the same as the previous one, spatial variation of mean word age will merely correspond to a slight difference of words among peripheral populations. This is particularly crucial in applying the model to different types of variants beyond lexicons, as different linguistic features are reported to evolve at various rates [34,35]. To clarify this, future work could incorporate the linguistic features and mutation into our model, and a possible mathematical framework is the 0,1-vector model [36]. As the number of mutation events through the diffusion process can be considered proportional to the word age, our model may be extended to calculate the spatial pattern of the amount of accumulating mutation. In this way, it may be possible to quantify the distribution of linguistic features and calculate the similarity or difference of culture among populations.

## Supplementary Material

online supplementary.docx

## Supplementary Material

figure 1d(1).nb

## Supplementary Material

figure 1d(2).nb

## Supplementary Material

figure 1e(1).nb

## Supplementary Material

figure 1e(2).nb

## Supplementary Material

figure 2b,c(1).nb

## Supplementary Material

figure 2b,c(2).nb

## Supplementary Material

figure 2b,c(3).nb

## Supplementary Material

figure 3c.nb

## Supplementary Material

figure 3d.nb

## Supplementary Material

figure 4a,b.nb

## Supplementary Material

figure 5 mean_age,gamma=10.xlsx

## Supplementary Material

figure 5 mean_age,gamma=20.xlsx

## Supplementary Material

figure 5 mean_age,gamma=50.xlsx

## Supplementary Material

instruction of supplementary files.txt

## Data accessibility

Mathematica files that we used for numerical analysis are included in the electronic supplementary material.

## References

[1] National Institute for Japanese Language and Linguistics. 1966–1974 Linguistic Atlas of Japan 1–6.

[2] Orton H, Sanderson S, Widdowson J 1998 *The linguistic atlas of England* London, UK: Routledge.

[3] Gilliéron J, Edmont E 1903–1910 *Atlas linguistique de la France* Paris, France: Champion.

[4] Gray RD, Drummond AJ, Greenhill SJ 2009 Language phylogenies reveal expansion pulses and pauses in Pacific settlement. Science 323, 479–483. (10.1126/science.1166858) 19164742

[5] Bouckaert R et al 2012 Mapping the origins of the Indo-European language family. Science 337, 957–960. (10.1126/science.1219669) 22923579 PMC4112997

[6] Grollemund R, Branford S, Bostoen K, Meade A, Venditti C, Pagel M 2015 Bantu expansion shows that habitat alters the route and pace of human dispersals. Proc. Natl Acad. Sci. USA 112, 13 296–13 301. (10.1073/pnas.1503793112) PMC4629331 26371302

[7] Mesoudi A 2011 Cultural evolution: how Darwinian theory can explain human culture & synthesize the social sciences. Chicago, IL: University of Chicago Press.

[8] Jeszenszky P, Hikosaka Y, Imamura S, Yano K 2019 Japanese lexical variation explained by spatial contact patterns. ISPRS Int. J. Geo-Inf. 8, 400 (10.3390/ijgi8090400)

[9] Szmrecsanyi B 2012 Geography is overrated. In Dialectological and folk dialectological concepts of space (eds Hansen S, Schwarz C, Stoeckle P, Streck T), pp. 215–231. Berlin, Germany: De Gruyter.

[10] Yanagita K 1927 Kagyuko (1)-(4). Jinruigaku Zasshi 42, 125–135 (162–172, 223–233, 273–284).

[11] Blanchard N 2008 Dialectologie et standarisation linguistique—Centres et Marges économiques et culturels en Basse-Bretagne. Port Acadie, (13-14-15), 45–61.

[12] Le Roux P 1924–1953 *Atlas Linguistique de la Basse-Bretagne* Paris, France: Champion.

[13] Trudgill P 1974 Linguistic change and diffusion: description and explanation in sociolinguistic dialect geography. Lang. Soc. 2, 215–246. (10.1017/S0047404500004358)

[14] Kretzschmar WA Jr, Jusso I, Bailey CT 2014 Computer simulation of dialect feature diffusion. J. Linguist. Geogr. 2, 41–57. (10.1017/jlg.2014.2)

[15] Fagyal Z, Swarup S, Escobar AM, Gasser L, Lakkaraju K 2010 Centers and peripheries: network roles in language change. Lingua 120, 2061–2079. (10.1016/j.lingua.2010.02.001)

[16] Burridge J 2017 Spatial evolution of human dialects. Phys. Rev. X 7, 031008 (10.1103/PhysRevX.7.031008)

[17] Burridge J 2018 Unifying models of dialect spread and extinction using surface tension dynamics. R. Soc. Open Sci. 5, 171446 (10.1098/rsos.171446) 29410847 PMC5792924

[18] Lizana L, Mitarai N, Kim S 2011 Modeling the spatial dynamics of culture spreading in the presence of cultural strongholds. Phys. Rev. E 83, 066116 (10.1103/PhysRevE.83.066116) 21797450

[19] Matsumoto O 1993 Zenkoku aho-baka bumpu-kou. Tokyo, Japan: Ohta Shuppan.

[20] Inoue F 2009 Year of first attestation of standard Japanese forms and gravity centre by railway distance. Dialectol. Geolinguist. 17, 118–123. (10.1515/DIG.2009.007)

[21] Geospatial Information Authority of Japan. See https://www.gsi.go.jp/KOKUJYOHO/kenchokan.html (accessed 20 November 2019).

[22] Statistics Bureau of Japan. 2018 See https://www.e-stat.go.jp/stat-search/files?page=1&layout=datalist&toukei=00200524&tstat=000000090001&cycle=7&year=20180&month=0&tclass1=000001011679&stat_infid=000031807142 (accessed 4 March 2020).

[23] Cavalli-Sforza LL, Feldman MW 1981 Cultural transmission and evolution: a quantitative approach. Princeton, NJ: Princeton University Press. 7300842

[24] Boyd R, Richerson PJ 1985 Culture and the evolutionary process. Chicago, IL: University of Chicago Press.

[25] Mace R, Holden CJ, Shennan S 2005 The evolution of cultural diversity: a phylogenetic approach. London, UK: University College London Press.

[26] Nunn CL, Mulder MB, Langley S 2006 Comparative methods for studying cultural trait evolution: a simulation study. Cross Cult. Res. 40, 177–209. (10.1177/1069397105283401)

[27] Currie TE, Greenhill SJ, Gray RD, Hasegawa T, Mace R 2010 Rise and fall of political complexity in island South-East Asia and the Pacific. Nature 467, 801–804. (10.1038/nature09461) 20944739

[28] Towner MC, Grote MN, Venti J, Mulder MB 2012 Cultural macroevolution on neighbor graphs. Vertical and horizontal transmissions among western north American Indian societies. Hum. Nat. 23, 283–305. (10.1007/s12110-012-9142-z) 22791406

[29] Brown S, Savage PE, Ko AMS, Stoneking M, Ko YC, Loo JH, Trejaut AJ 2014 Correlations in the population structure of music, genes and language. Proc. R. Soc. B 281, 20132072 (10.1098/rspb.2013.2072) PMC3843827 24225453

[30] Savage PE, Brown S 2014 Mapping music: cluster analysis of song-type frequencies within and between cultures. Ethnomusicology 58, 133–155. (10.5406/ethnomusicology.58.1.0133)

[31] Tamura K, Ihara Y 2017 Quantifying cultural macro-evolution: a case study of hinoeuma fertility drop. Evol. Hum. Behav. 38, 117–124. (10.1016/j.evolhumbehav.2016.07.007)

[32] Dybiec B, Mitarai N, Sneppen K 2012 Information spreading and development of cultural center. Phys. Rev. E 85, 056116 (10.1103/PhysRevE.85.056116) 23004830

[33] Nakamura D 2011 The diversity of mortuary practice acceptance at the beginning of the Yayoi Period. In Coexistence and cultural transmission in East Asia (eds Matsumoto N, Bessho H, Tomii M), pp. 223–256. Walnut Creek, CA: Left Coast Press.

[34] Greenhill SJ, Wu CH, Hua X, Dunn M, Levinson SC, Gray RD 2017 Evolutionary dynamics of language systems. Proc. Natl Acad. Sci. USA 114, E8822–E8829. (10.1073/pnas.1700388114) 29073028 PMC5651730

[35] Pagel M, Meade A. 2017 The deep history of the number words. Phil. Trans. R. Soc. B 373, 20160517 (10.1098/rstb.2016.0517) 29292363 PMC5784043

[36] Fogarty L, Wakano JY, Feldman MW, Aoki K 2017 The driving forces of cultural complexity. Neanderthals, modern humans, and question of population size. Hum. Nat. 28, 39–52. (10.1007/s12110-016-9275-6) 27783325

