# Supplementary material for: Quantifying the spatial pattern of dialect words spreading from a central population
Source: J R Soc Interface. 2020 Jul 8;17(168):20200335. doi: 10.1098/rsif.2020.0335 (PMC7423420; doi:10.1098/rsif.2020.0335)

Quantifying the spatial pattern of dialect words spreading from a central population

Takuya Takahashi and Yasuo Ihara

**Online Supplementary Material**

This file consists of three sections: “Distribution of cultural age”, “Derivation of the formulae in one-dimensional diffusion”, and “Agent-based simulation on infinite populations”. We also provide an appendix at the end of the document.

*Distribution of cultural age*

In this section, we elaborate a more detailed derivation of frequency distribution, mean value, and the standard deviation of word age.

From (2.2) and (2.3), when $\rho\geq2$, we have

$$f_{k}\left( \rho,t \right)=\sum_{j=1}^{n} a_{kj}f_{j}\left( \rho-1,t-1 \right) \left( 1\leq k\leq n, \rho\geq2 \right). (S1)$$

Defining $\boldsymbol{f}\left( \rho,t \right)=\left( f_{1}\left( \rho,t \right)\ldots f_{n}\left( \rho,t \right) \right)^{T}$, $\boldsymbol{A}=\left( \begin{matrix} a_{11} & \cdots& a_{1n} \\ \vdots& \ddots& \vdots\\ a_{n1} & \cdots& a_{nn} \end{matrix} \right)$, we have

$$\boldsymbol{f}\left( \rho,t \right)=\boldsymbol{Af}\left( \rho-1,t-1 \right) \left( \rho\geq2 \right). (S2)$$

By using (S2), we calculate the age frequency for peripheral populations. Since words with age zero cannot exist in any population other than the central population, we have

$$\boldsymbol{f}\left( 0,t \right)=\boldsymbol{0,} (S3)$$

where $\boldsymbol{0}$ is the *n*-dimensional zero vector. For variants which are created after the emergence of central population at $t=0$, we have

$$\boldsymbol{f}\left( \rho,t \right)=\boldsymbol{A}^{\rho-1}\boldsymbol{f}\left( 1,t-\rho+1 \right)=\boldsymbol{A}^{\rho-1}\left( \begin{matrix} a_{10} \\ \vdots\\ a_{n0} \end{matrix} \right) \left( 1\leq\rho\leq t \right). (S4)$$

As for the variants which date back before the emergence of central population, we have

$$\boldsymbol{f}\left( \rho,t \right)=\boldsymbol{A}^{t}\boldsymbol{f}\left( \rho-t,0 \right) \left( \rho>t \right), (S5)$$

where $\boldsymbol{f}\left( \rho,0 \right)$ represents the initial word-age distribution in the peripheral populations. Here, (S3), (S4), and (S5) represent the age distribution of words. Using these equations,

$$\boldsymbol{r}\left( t \right)=\sum_{\rho=0}^{\infty} \rho\boldsymbol{f}\left( \rho,t \right)=\sum_{\rho=1}^{t} \rho\boldsymbol{A}^{\rho-1}\left( \begin{matrix} a_{10} \\ \vdots\\ a_{n0} \end{matrix} \right)+\sum_{\rho=t+1}^{\infty} \rho\boldsymbol{A}^{t}\boldsymbol{f}\left( \rho-t,0 \right)=\sum_{\rho=1}^{t} \rho\boldsymbol{A}^{\rho-1}\left( \begin{matrix} a_{10} \\ \vdots\\ a_{n0} \end{matrix} \right)+\boldsymbol{A}^{t}\boldsymbol{r}\left( 0 \right)+t\boldsymbol{A}^{t}\left( \begin{matrix} 1 \\ \vdots\\ 1 \end{matrix} \right). (S6)$$

Defining the *n*-dimensional square matrix ***S*** by

$$\boldsymbol{S}=\sum_{\rho=1}^{t} \rho\boldsymbol{A}^{\rho-1}, (S7)$$

we have

$$\boldsymbol{SA}=\sum_{\rho=1}^{t} \rho\boldsymbol{A}^{\rho}=\sum_{\rho=2}^{t+1} \left( \rho-1 \right)\boldsymbol{A}^{\rho-1}. (S8)$$

Subtracting (S8) from (S7),

$$\boldsymbol{S}\left( \boldsymbol{E-A} \right)=\boldsymbol{E}+\sum_{\rho=2}^{t} \boldsymbol{A}^{\rho-1}-t\boldsymbol{A}^{t}=\boldsymbol{E}+\boldsymbol{A}\left( \boldsymbol{E}-\boldsymbol{A}^{t-1} \right)\left( \boldsymbol{E}-\boldsymbol{A} \right)^{-1}-t\boldsymbol{A}^{t}=\left( \boldsymbol{E}-\boldsymbol{A}^{t} \right)\left( \boldsymbol{E}-\boldsymbol{A} \right)^{-1}-t\boldsymbol{A}^{t}. (S9)$$

Hence, we have

$$\boldsymbol{S=}\sum_{\rho=1}^{t} \rho\boldsymbol{A}^{\rho-1}=\left[ \left( \boldsymbol{E}-\boldsymbol{A}^{t} \right)\left( \boldsymbol{E}-\boldsymbol{A} \right)^{-1}-t\boldsymbol{A}^{t} \right]\left( \boldsymbol{E}-\boldsymbol{A} \right)^{-1}. (S10)$$

Considering (2.4),

$$\left( \boldsymbol{E-A} \right)\left( \begin{matrix} 1 \\ \vdots\\ 1 \end{matrix} \right)=\left( \begin{matrix} 1-\left( a_{11}+\cdots+a_{1n} \right) \\ \vdots\\ 1-\left( a_{n1}+\cdots+a_{nn} \right) \end{matrix} \right)=\left( \begin{matrix} a_{10} \\ \vdots\\ a_{n0} \end{matrix} \right), (S11)$$

which gives

$$\left( \boldsymbol{E}-\boldsymbol{A} \right)^{-1}\left( \begin{matrix} a_{10} \\ \vdots\\ a_{n0} \end{matrix} \right)=\left( \begin{matrix} 1 \\ \vdots\\ 1 \end{matrix} \right). (S12)$$

Substituting (S10) for (S6) and using (S12), the mean word age is given by

$$\boldsymbol{r}\left( t \right)=\left[ \left( \boldsymbol{E}-\boldsymbol{A}^{t} \right)\left( \boldsymbol{E}-\boldsymbol{A} \right)^{-1}-t\boldsymbol{A}^{t} \right]\left( \begin{matrix} 1 \\ \vdots\\ 1 \end{matrix} \right)+\boldsymbol{A}^{t}\boldsymbol{r}\left( 0 \right)+t\boldsymbol{A}^{t}\left( \begin{matrix} 1 \\ \vdots\\ 1 \end{matrix} \right)=\boldsymbol{A}^{t}\left[ \boldsymbol{r}\left( 0 \right)-\left( \boldsymbol{E}-\boldsymbol{A} \right)^{-1}\left( \begin{matrix} 1 \\ \vdots\\ 1 \end{matrix} \right) \right]+\left( \boldsymbol{E}-\boldsymbol{A} \right)^{-1}\left( \begin{matrix} 1 \\ \vdots\\ 1 \end{matrix} \right). (S13)$$

Now we consider the equilibrium state. (S3), (S4), and (S5) give

$$\boldsymbol{f}\left( \rho,\infty\right)=\left\{ \begin{aligned} \boldsymbol{0}\mathrm{if} \rho=0 \\ \boldsymbol{A}^{\rho-1}\left( \begin{matrix} a_{10} \\ \vdots\\ a_{1n} \end{matrix} \right) \mathrm{otherwise} \end{aligned}, \right. (S14)$$

which in turn gives

$$\boldsymbol{r}\left( \infty\right)=\sum_{\rho=0}^{\infty} \rho\boldsymbol{f}\left( \rho,\infty\right)=\left( \boldsymbol{E}-\boldsymbol{A} \right)^{-1}\left( \begin{matrix} 1 \\ \vdots\\ 1 \end{matrix} \right), (S15)$$

$$\boldsymbol{q}\left( \infty\right)=\sum_{\rho=0}^{\infty} \rho^{2}\boldsymbol{f}\left( \rho,\infty\right)=\left( \boldsymbol{E}-\boldsymbol{A} \right)^{-1}\left( \boldsymbol{E}-\boldsymbol{A} \right)^{-1}\left( \boldsymbol{E}+\boldsymbol{A} \right)\left( \begin{matrix} 1 \\ \vdots\\ 1 \end{matrix} \right), (S16)$$

where $\boldsymbol{q}\left( t \right)=\left( q_{1}\left( t \right)\cdots q_{n}\left( t \right) \right)^{T}$ denotes the second-order moment, from which the variance of word age, $\boldsymbol{v}\left( t \right)=\left( v_{1}\left( t \right)\cdots v_{n}\left( t \right) \right)^{T}$, is obtained. Standard deviation of word age $\boldsymbol{\sigma}\left( t \right)=\left( \sigma_{1}\left( t \right)\cdots\sigma_{n}\left( t \right) \right)^{T}$is readily obtained by taking the square root of the variance. Note that every absolute value of eigenvalue of $\boldsymbol{A}$ is less than 1 (see **Appendix**), so that the infinite series in (S15) and (S16) converge.

*Derivation of the formulae in one-dimensional diffusion*

We derive the formulae to compute the mean and standard deviation of cultural age in one-dimensional unidirectional diffusion (i.e., (2.9) for unidirectional, (2.11) for bidirectional diffusion).

As for the unidirectional diffusion model, (2.9) gives

$$\left( \boldsymbol{E}-\boldsymbol{A} \right)^{-1}=\frac{1}{a}\left( \begin{matrix} 1 & & \\ \vdots& \ddots& \\ 1 & \ldots& 1 \end{matrix} \right). (S17)$$

Thus, (S15) and (S16) give

$$r_{k}\left( \infty\right)=\frac{k}{a}, (S18)$$

$$q_{k}\left( \infty\right)=\frac{k^{2}}{a^{2}}+\frac{k}{a^{2}}-\frac{k}{a}. (S19)$$

We can calculate the standard deviation by

$$\sigma_{k}\left( \infty\right)=\sqrt{q_{k}\left( \infty\right)-{r_{k}\left( \infty\right)}^{2}}=\sqrt{\frac{k}{a}\left( \frac{1}{a}-1 \right)}. (S20)$$

In the case of bidirectional diffusion, (2.11) gives

$$\left( \boldsymbol{E}-\boldsymbol{A} \right)^{-1}=\frac{1}{a}\left( \begin{matrix} 1 & 1 & 1 & \cdots& 1 \\ 1 & 2 & 2 & \cdots& 2 \\ 1 & 2 & 3 & \cdots& 3 \\ \vdots& \vdots& \vdots& \ddots& \vdots\\ 1 & 2 & 3 & \cdots& n \end{matrix} \right). (S21)$$

Thus, using (S15) and (S16), we have

$$r_{k}\left( \infty\right)=\frac{k}{2a}\left( 2n-k+1 \right), (S22)$$

$$q_{k}\left( \infty\right)=\frac{k}{12a^{2}}\left\{ \left( k^{2}-1 \right)\left( k-4n-2 \right)+4n\left( n+1 \right)\left( 2n+1 \right) \right\}-\frac{k}{2a}\left( 2n-k+1 \right). (S23)$$

Hence, we have

$$\sigma_{k}\left( \infty\right)=\sqrt{q_{k}\left( \infty\right)-{r_{k}\left( \infty\right)}^{2}}=\sqrt{\frac{k}{6a^{2}}\left( 2n-k+1 \right)\left( 2n^{2}-2nk+k^{2}+2n-k+1 \right)-\frac{k}{2a}\left( 2n-k+1 \right)}. (S24)$$

*Agent-based simulation on infinite populations*

We have assumed that each population in the network is composed of infinite number of individuals. Under this assumption, the effect of stochasticity is absent, and we can derive the mean word age in a deterministic manner. In contrast, this section deals with an agent-based finite-individual model and simulates how the mean word age fluctuates due to cultural drift.

We use the one-dimensional unidirectional model with $n=5$ (i.e., chain of one central population and five peripheral populations). Each peripheral population consists of $N$ individuals. We do not specify the population size of the central population because it does not affect the result. In each timestep, each new-born individual in the peripheral populations learns one variant from the previous timestep. An individual in $P_{k} \left( k\neq1 \right)$ learns from $P_{k-1}$ with probability $a$ and learns from $P_{k}$ otherwise. (S)he learns from an arbitrary individual (role model) in the chosen population with probability $1/N$ for each. When (s)he observes a word aged $\rho$ in the immediate timestep, the age of the acquired variant becomes $\rho+1$ because one timestep has passed. In particular, those who learn from the central population obtain the variant with age $1$.

We started the simulation by the initial state where every individual has the variant aged one and measured the mean word age in five populations for 2000 timesteps. After discarding the first 1000 timesteps to eliminate the bias of initial state, we calculated the average and standard deviation of the mean word age over the last 1000 timesteps. Figure 7a illustrates changes in the mean word ages across 2000 timesteps in a single simulation run, indicating that mean word age fluctuates over time due to stochastic effect. Nevertheless, the mean word ages obtained by the deterministic model are in good accordance with the simulation results, specifically, the mean word ages averaged over 1000 timesteps, even when the populations are considerably small (Figure 7b). Figure 7b also shows that the standard deviation of the mean word age over 1000 timesteps increases with the distance from the central population (Figure 7b). In addition, the standard deviation decreases with increasing population size. Therefore, the mean word age in each timestep will become closer to the analytically obtained result (i.e., equation (10a)).

**Appendix**

*On the eigenvalues of* ***A***

Here we prove the absolute value of every eigenvalue of transmission matrix $\boldsymbol{A}$ is smaller than 1.

Let $\lambda$ be any eigenvalue of $\boldsymbol{A}$, and an eigenvector corresponding to $\lambda$ is denoted by $\boldsymbol{p}=\left( \begin{matrix} p_{1} & \ldots& p_{n} \end{matrix} \right)^{T}\neq\boldsymbol{0}$. We define $p_{k}$ as the element of $\boldsymbol{p}$, such that $\left| p_{k} \right|=\max_{j} \left| p_{j} \right|.$ As $\lambda\boldsymbol{p}=\boldsymbol{Ap}$, we have

$$\lambda p_{k}=\sum_{j=1}^{n} a_{kj}p_{j}. (A1)$$

Therefore,

$$\left| \lambda\right|\left| p_{k} \right|\leq\sum_{j=1}^{n} a_{kj}\left| p_{j} \right|\leq\sum_{j=1}^{n} a_{kj}\left| p_{k} \right|=\left( 1-a_{k0} \right)\left| p_{k} \right|\leq\left| p_{k} \right|. (A2)$$

As $\left| p_{k} \right|>0$,we have$\left| \lambda\right|\leq1.$

Now we will disprove the case $\left| \lambda\right|=1$. Assuming $\left| \lambda\right|=1$, (A2) gives

$$a_{k0}=0, \mathrm{and} a_{kj}\left( \left| p_{k} \right|-\left| p_{j} \right| \right)=0 for any j \left( 1\leq j\leq n \right). (A3)$$

There exists an array of integers $C_{0},\ldots,C_{l}$, such that

$$C_{0}=0, C_{l}=k, \mathrm{and} a_{C_{i}C_{i-1}}>0. (A4)$$

Substituting $c_{l-1}$ into $j$ in the second expression of (A3), we have $\left| p_{c_{l-1}} \right|=\left| p_{c_{l}} \right|=\max_{j} \left| p_{j} \right|$. This in turn gives $a_{c_{l-1}0}=0$ and $\left| p_{c_{l-2}} \right|=\max_{j} \left| p_{j} \right|$ with the same discussion as (A2). Similarly, we can conclude

$$a_{c_{l}0}=a_{c_{l-1}0}=\ldots=a_{c_{1}0}=0 (A5)$$

by mathematical induction. However, $a_{c_{1}0}=0$ contradicts the expressions (A4) for $i=1$. Therefore, $\left| \lambda\right|<1.$

Figure legend

Fig.7

Changes in the mean word ages over 2000 generations in five populations in a simulation assuming a finite population size. Unidirectional transmission in the one-dimensional model is considered. (a) $N=1000.$ Fluctuation of mean word age in $P_{1}$ (blue), $P_{2}$ (red), $P_{3}$ (gray), $P_{4}$ (yellow), and $P_{5}$ (green). (b) Average over the last 1000 generations of mean cultural age. Blue, red and gray bars represent the case $N=100, 1000, and 5000$, respectively. Yellow bar represents the theoretical value of the infinite-individual model obtained by (10a). Error bar indicates standard deviation. Parameter value: $a=0.1$.

Fig. 7


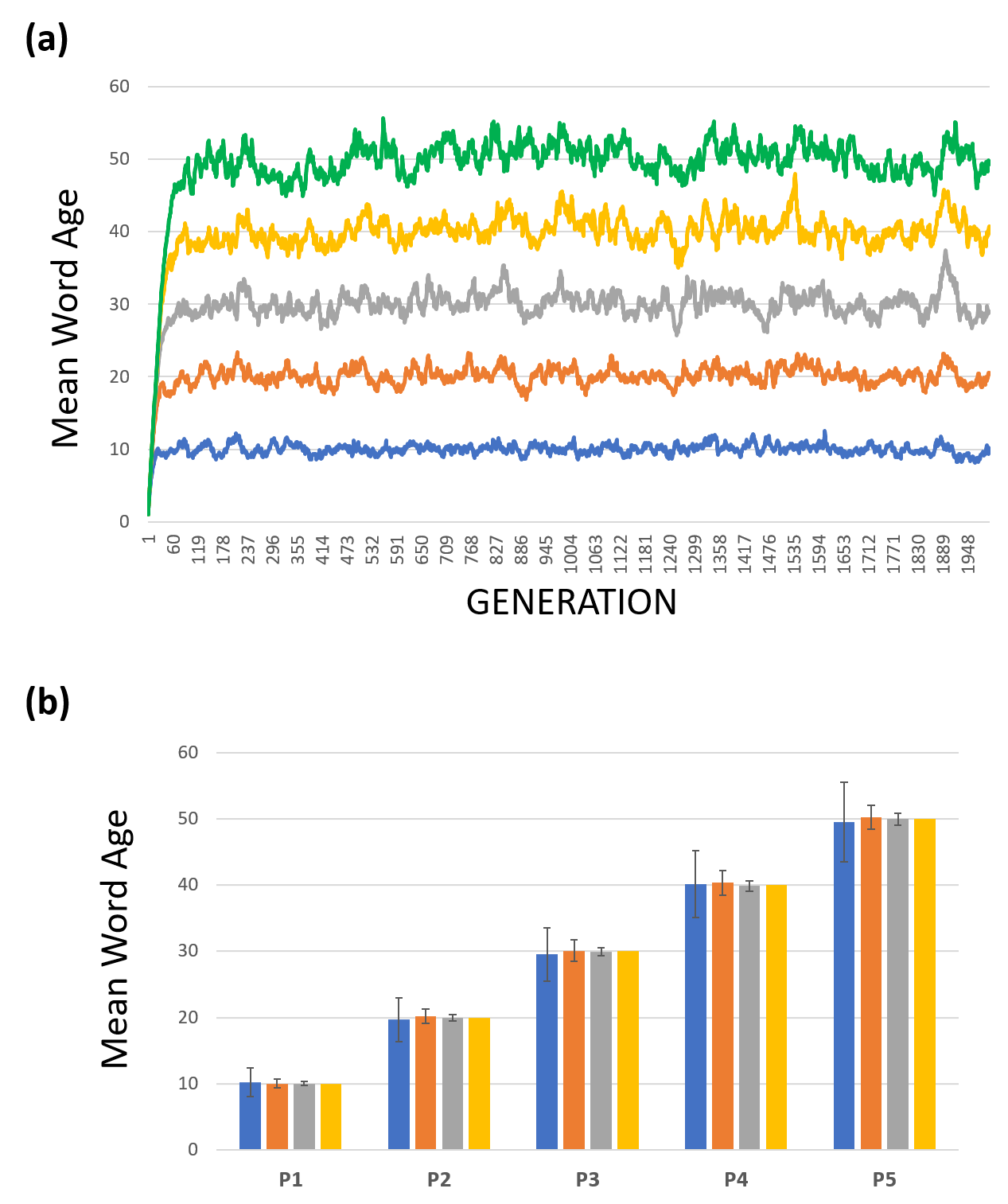

Supplement: online supplementary.docx [file rsif20200335supp1.docx]
